# Chronic stress and energy homoeostasis

**DOI:** 10.18632/aging.102500

**Published:** 2019-11-20

**Authors:** Chi Kin Ip, Herbert Herzog, Lei Zhang

**Affiliations:** 1 Neuroscience Division, Garvan Institute of Medical Research, Darlinghurst, Sydney NSW 2010, Australia; 2 Faculty of Medicine, University of New South Wales, Sydney NSW 2052, Australia

**Keywords:** feeding, stress, obesity, NPY, amygdale

Stress is commonplace to modern society and can be a major factor negatively influencing a balanced lifestyle which is crucial to maintain a healthy aging process. While acute stress can be beneficial for dealing with unexpected situations, prolonged stress has been shown to have adverse consequences on many organ systems as well as negative impacts on behavioural and physiological responses including feeding behaviour and energy homeostasis. In humans, stress affects individuals differently with regards to feeding; some decrease food intake and lose weight during or after stress, while most others increase their food intake during stress. Importantly, however, independent of hypo- or hyperphagia, people often report that they prefer highly palatable food during periods of prolonged stress, rich in both sugar and fat, which significantly contributes to the development of obesity. Similarly, when rodents have a choice of highly palatable diet, stress also increases the intake of such calorie dense foods [1]. Despite these well documented observations in both humans and animals the underlying neuronal pathways that are responsible for controlling this behaviour are still unclear.

Central control of energy homeostasis is known to be orchestrated by a complex neuronal network with neuropeptide Y (NPY)-ergic pathways being one of the most prominent. NPY is a 36-amino acid neuropeptide that is highly expressed in the arcuate nucleus of the hypothalamus (Arc), a brain region critical in the regulation of energy homeostasis. However, recent study also points to an important role of NPY in aging and lifespan determination, with lack of NPY in mice leading to loss of the beneficial effects under calorie restriction which would otherwise confer on lifespan extension [2]. Negative energy balance leads to an increase in Arc-NPY level, which activates energy-conservation pathways, accompanied by hyperphagia and reduction in energy expenditure, allowing the re-establishment of energy balance. Apart from the Arc, NPY-expressing neurons are also found at a considerably high level in the medial nuclei of the central amygdala (CeA), a region known to be critical in controlling anxiety and stress related behaviours [3]. However, the involvement of CeA activity in the control of energy homeostasis had not been explored. In a recent study we have now demonstrated that CeA-NPY neurons play a pivotal role in the regulation of energy metabolism, especially under chronic stress conditions [4]. Acute activation of CeA-NPY neurons by using chemogenetic tools is sufficient to trigger intrinsic food intake and lower energy expenditure, which is similar to that seen with Arc-NPY neurons activation [4]. More importantly, this population of CeA-NPY neurons is instrumental for chronic stress-induced increases in palatable food consumption and weight gain. Under a chronic stress paradigm combined with a high-fat diet (HFD), mice developed exacerbated obesity due to an increase in caloric intake and a concomitant reduction in energy expenditure, whereas mice stressed but fed a normal chow diet exhibited a reduction in caloric intake with a compensatory reduction in energy expenditure resulting in an overall unaltered body weight [4]. Interestingly, an increase in CeA-NPY expression was seen in the stressed mice on HFD feeding, but not on chow. Using a viral approach to manipulate NPY expression in CeA-NPY neurons under the combined stress and HFD condition we further demonstrated that the lack of NPY selectively in CeA neurons can attenuate this obese phenotype, while overproduction of NPY in the CeA exacerbates it, both through dual effects on food intake and energy expenditure [4].

Circulating hormones are known to signal information on peripheral energy status either via vagal connections or directly by accessing their respective receptors via various circumventricular organs to the brain [5]. Two of the main hormones involved in this process are insulin and leptin. Interestingly, within the CeA only Insulin receptors (InsR), but not leptin receptors (LeprR) can be found on *Npy*-expressing neurons, consistent with our finding that insulin infusion into the CeA shows a dosage-dependent inhibition of *Npy* levels [4]. Importantly, this downregulation of *Npy* by insulin on CeA neurons was lost under chronic stress combined with HFD condition. We further showed that ablation of the InsR specifically only in CeA-NPY neurons recapitulated the hyperphagic and exacerbated obese phenotype in mice, similarly to that observed in those with CeA-NPY overproduction when under stress combined with HFD condition, suggesting that the loss of responsiveness of CeA-NPY neurons to insulin signalling is the primary cause for the food over-consummatory behaviour in these mice [4].

In addition to the NPY circuit, other orexigenic neuron populations also exist in the CeA. For instance, GABAergic serotonin receptor 2a (*Htr2a*)-expressing CeA neuron activation promotes intrinsic food consummatory behaviour through a positive-valence mechanism [6]. Similarly, activation of prepronociceptin-expressing CeA neurons enhances palatable food intake via promoting the rewarding properties of the palatable food [7]. Whether and to what extend these orexigenic CeA neurons co-express NPY and the functional differences among these neuronal subsets remain to be evaluated. In addition to these orexigenic neuron populations, CeA GABAergic cells that express protein kinase C- δ (Pkc-δ) have been shown to exert a strong anorexigenic effect and suggested to act as a central node integrating different anorexigenic signals [8]. Importantly, mapping studies revealed an intricate reciprocal network within the CeA among the circuits involved in feeding regulation. Considering NPY levels decline with age, it is conceivable that the interactions among these feeding CeA circuits evolve with aging process. The mechanisms underlying these interactions and how they evolve with aging process and ultimately impact on the way energy balance is managed under stress condition warrant future research.
